# Supplementary material for: Molecularly distinct models of zebrafish Myc-induced B cell leukemia
Source: Leukemia. 2018 Dec 20;33(2):559–62. doi: 10.1038/s41375-018-0328-1 (PMC6365381; doi:10.1038/s41375-018-0328-1)
Supplement: Supplementary file 1 — Supplemental Material [file 41375_2018_328_MOESM1_ESM.pdf]

Supplementary Materials for

# Molecularly distinct models of zebrafish *Myc*-induced B cell leukemia

Chiara Borga[1*], Clay A. Foster[1*], Sowmya Iyer[2,3*], Sara P. Garcia, David M. Langenau[2,3]†, J. Kimble Frazer[1]†

Correspondence to: Kimble-Frazer@ouhsc.edu; dlangenau@mgh.harvard.edu

## Methods

### RNA-seq of *hMYC* ALL samples

Zebrafish were housed in an aquatic colony at 28.5°C on a 14:10 hour light:dark circadian cycle and cared for according to protocols approved by the University of Oklahoma Health Sciences Center IACUC (12-066 and 15-046). ALL were identified in 6 month old *rag2*:*hMYC*; *lck*:*eGFP* zebrafish of both genders using fluorescence microscopy, with RNA extracted from FACS-purified GFP$^+$ lymphocytes as described previously[1]. Libraries were constructed from 100-700 ng total RNA according to the TruSeq Stranded mRNA sample preparation protocol (Illumina, San Diego, CA), sequenced using an Illumina Hi-Seq 3000, and base calling performed using Illumina bcl2fastq2 software. Paired-end sequencing of 100 or 150 bp reads were conducted on *hMYC* ALLs. RNA-seq data are deposited at NCBI GEO repository GSE119173 (https://www.ncbi.nlm.nih.gov/geo/query/acc.cgi?acc=GSE119173; enter security token "izqfoyyslditlgl" where indicated). Newly generated *hMYC* RNA sequencing data was compared with *mMyc* RNA sequencing data available at GSE108855.

### RNA-seq alignment and analysis

Alignment and RNA-seq read counting, identification of human orthologues, and clonality analysis were performed as described by Garcia et al[2]. For data in Figure 2B, differentially-expressed genes were first identified in a pairwise manner using DESeq2. Genes were further

filtered as follows: (a) log2 fold-change in each foreground category compared to each of the other two background categories >1, with an adjusted p-value <0.05, and (b) absolute expression in reads/million >100 in the foreground category in at least 16 out of 20 T-ALL samples, or at least two samples for each the *ighz*[+] or *ighm*[+] B-ALLs.

## References


1. Borga C, Park G, Foster C, Burroughs-Garcia J, Marchesin M, Shah R, *et al.* Simultaneous B and T cell acute lymphoblastic leukemias in zebrafish driven by transgenic MYC: implications for oncogenesis and lymphopoiesis. *Leukemia* 2018 2018/08/15.

2. Garcia EG, Iyer S, Garcia SP, Loontiens S, Sadreyev RI, Speleman F, *et al.* Cell of origin dictates aggression and stem cell number in acute lymphoblastic leukemia. *Leukemia* 2018 2018/04/18.
